# Mechanisms of immune checkpoint inhibitors: insights into the regulation of circular RNAS involved in cancer hallmarks

**DOI:** 10.1038/s41419-023-06389-5

**Published:** 2024-01-04

**Authors:** Lingjiao Meng, Haotian Wu, Jiaxiang Wu, Ping’an Ding, Jinchen He, Meixiang Sang, Lihua Liu

**Affiliations:** 1 Department of Tumor Immunotherapy, The Fourth Hospital of Hebei Medical University, Shijiazhuang, Hebei 050035 China; 2 Research Center and Tumor Research Institute, The Fourth Hospital of Hebei Medical University, Shijiazhuang, Hebei 050017 China; 3 The Third Department of Surgery, The Fourth Hospital of Hebei Medical University, Shijiazhuang, Hebei, 050011 China; 4 Hebei Key Laboratory of Precision Diagnosis and Comprehensive Treatment of Gastric Cancer, Shijiazhuang, 050011 China; 5 Science and Education Department, Shanghai Electric Power Hospital, Shanghai, 20050 China

**Keywords:** Cancer epidemiology, Non-coding RNAs

## Abstract

Current treatment strategies for cancer, especially advanced cancer, are limited and unsatisfactory. One of the most substantial advances in cancer therapy, in the last decades, was the discovery of a new layer of immunotherapy approach, immune checkpoint inhibitors (ICIs), which can specifically activate immune cells by targeting immune checkpoints. Immune checkpoints are a type of immunosuppressive molecules expressed on immune cells, which can regulate the degree of immune activation and avoid autoimmune responses. ICIs, such as anti-PD-1/PD-L1 drugs, has shown inspiring efficacy and broad applicability across various cancers. Unfortunately, not all cancer patients benefit remarkably from ICIs, and the overall response rates to ICIs remain relatively low for most cancer types. Moreover, the primary and acquired resistance to ICIs pose serious challenges to the clinical application of cancer immunotherapy. Thus, a deeper understanding of the molecular biological properties and regulatory mechanisms of immune checkpoints is urgently needed to improve clinical options for current therapies. Recently, circular RNAs (circRNAs) have attracted increasing attention, not only due to their involvement in various aspects of cancer hallmarks, but also for their impact on immune checkpoints in shaping the tumor immune microenvironment. In this review, we systematically summarize the current status of immune checkpoints in cancer and the existing regulatory roles of circRNAs on immune checkpoints. Meanwhile, we also aim to settle the issue in an evidence-oriented manner that circRNAs involved in cancer hallmarks regulate the effects and resistance of ICIs by targeting immune checkpoints.

## Facts

- ICIs has achieved prominent efficacy in many types of cancer and revolutionized the status of cancer immunotherapy.
- Primary and acquired resistance limit the clinical application of ICIs in cancer immunotherapy.
- CircRNAs as emerging modulators of various cancer hallmarks are associated with the regulation of ICIs by targeting immune checkpoints.

## Questions

- Can circRNAs directly serve as immune checkpoints to regulate immune responses against cancer cells?
- Can circRNAs involved in cancer hallmarks such as proliferation and cell death mediate ICIs resistance?
- Is resistance to ICIs mediated by circRNAs in tumor cells or immune cells, or a combination of both?
- Can synthetic circRNAs regulate the cascades involved in immune checkpoint pathways and serve as new potential cancer immunotherapy regimens?

## Introduction

Cancer immunotherapy with ICIs by inhibition of negative immune regulation to enhance immune activity against cancer cells, which won the 2018 Nobel Prize in Physiology or Medicine, has revolutionized the treatment dilemma of various human malignancies [1–4]. The first immune checkpoint inhibitor approved by FDA named lpilimumab that targets cytotoxic T-lymphocyte-associated protein 4 (CTLA-4) to potentiate the T cell-mediated antitumor immune response, have remarkably improved the overall survival of metastatic melanoma patients [5–8]. Subsequently, the FDA approved programmed cell death protein 1 (PD-1) inhibitor nivolumab for the treatment of advanced melanoma due to its favorable overall survival, durable tumor remission after drug discontinuation, and acceptable long-term safety [9, 10]. Since then, the gate of immunotherapy has been thoroughly opened, and more monoclonal antibody drugs blocking PD-1 or its ligand PD-L1 have been approved in succession by the FDA alone or in combination with other drugs to treat numerous malignancies, such as melanoma, lung cancer, lymphoma, esophageal carcinoma, gastric cancer, and hepatocellular carcinoma [11–13]. Despite their prominent clinical efficacy, currently approved ICIs only benefit a subset of patients, even with response biomarkers like PD-L1 expression [14, 15]. The application of single-agent ICIs often leads to primary resistance, and some responsive patients develop acquired resistance to ICIs [16]. Additionally, immune-related adverse events (irAEs) owing to ICIs can induce excessive immune system activation or multiple organ disorders, and hyperprogression caused by ICIs pose the serious clinical challenges to immunotherapy [17–21]. Thus, identifying the molecular biological properties and regulatory mechanisms of immune checkpoints is crucial for optimizing therapeutic options and controlling adverse effects.

Immune checkpoints, a series of molecules that are expressed on immune cells and can tune up the degree of immune activation, act as gatekeepers during the body’s immune response to prevent overactivation of the immune system [22–24]. Over the past few decades, numerous immune checkpoint molecules have been identified, including but not limited to PD-1/PD-L1, CTLA-4, lymphocyte-activation gene 3 (LAG-3), T-cell immunoglobulin and mucin-domain containing-3 (TIM-3) [25]. Although the number of newly discovered immune checkpoints is considerable, the ICIs approved by FDA remain limited due to the complexity of their mechanisms. Recently, an increasing number of studies in molecular oncology have revealed the complicated regulatory mechanisms of immune checkpoint expression. Non-coding RNAs (ncRNAs), an abundant component of the human transcriptome involved in all cancer hallmarks, are highly correlated with the expression regulation of immune checkpoints [26–28]. One of the most unexpected discoveries in ncRNA field, with the advent of high-throughput sequencing techniques in recent years, was the identification of a new class of stable loop-structured molecules with both coding and non-coding properties, circRNAs [29]. They represent a novel category of transcripts that exhibit tissue- and spatio-temporal specific expression patterns, widely involved in multiple physiological and pathological processes [30]. Growing evidence has confirmed that circRNAs can serve as star molecules and play vital roles in cell signaling pathways regulation in human cancers [31–34]. During cancer progression, the body’s systemic immune landscape suffers from alterations in many aspects [35]. Meanwhile, the biological functions in anti-tumor immunity and potential applications in cancer immunotherapy have been gradually discovered [36–38]. However, circRNAs-mediated regulation of immune checkpoint molecules has not been well evaluated. Accordingly, this review will focus on the current immune checkpoints commonly applied in cancer and the molecular mechanisms by which circRNAs interfere with ICIs therapeutic strategies by regulating immune checkpoint pathways.

### Status quo of frequent immune checkpoints and ICIs in cancer

#### PD-1/PD-L1

PD-1/PD-L1 is the most well-characterized immune checkpoint pathway that has been widely employed in clinical cancer immunotherapy. PD-1 (also known as CD279), a member of CD28 superfamily, belongs to type I transmembrane glycoprotein with a single extracellular immunoglobulin variable domain, transmembrane region, and cytoplasmic domain containing immunoreceptor tyrosine-based inhibitory motif (ITIM) and immunoreceptor tyrosine-based switch motif (ITSM) [39]. It has been reported that PD-1 is a coinhibitory receptor, mainly expressed on activated T cells, but also on B cells, natural killer (NK) cells, myeloid cells, monocytes, neutrophils, and dendritic cells (DCs) [25, 40, 41]. PD-L1 (also known as CD274 or B7-H1), the dominant ligand of PD-1, belongs to the B7 family and can be expressed not only by immune cells, such as T cells, B cells, macrophages, DCs, and mast cells, but also by normal tissue cells and aberrant tumor cells [42, 43]. When PD-1 binds to PD-L1 on the surface of immune effector cells such as T cells, its cytoplasmic ITIM and ITSM can be phosphorylated, and then recruit Src homology 2 domain-containing tyrosine phosphatase 2 (SHP2) to further suppress T cell receptor (TCR) signaling transduction [44]. PD-1/PD-L1 pathway contributes to tumor immune escape, which has been identified as a main mechanism of adaptive immune resistance (AIR) to enable tumors resistant to immune response [45–47].

**Fig. 1:**
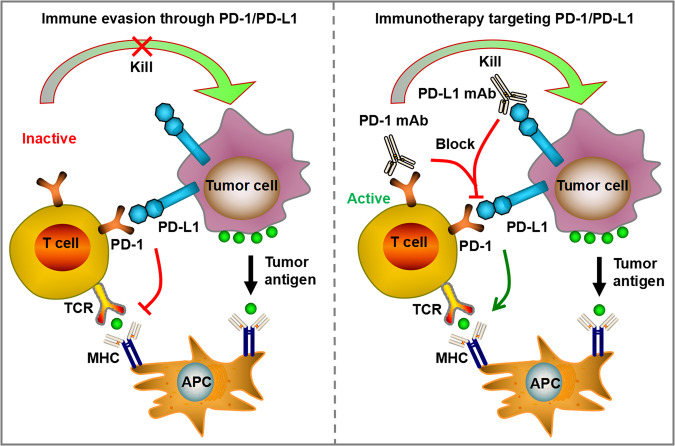
PD-1/PD-L1 pathway contributes to tumor immune escape, enabling tumors resistant to immune response. When PD-1 binds to PD-L1 on the surface of immune effector cells such as T cells, T cell receptor (TCR) signaling transduction was suppressed. Monoclonal antibodies (mAbs) blocking the PD-1/PD-L1 pathway have been widely applied for clinical immunotherapy to fight against a fraction of advanced cancer.

Monoclonal antibodies (mAbs) blocking the PD-1/PD-L1 pathway have been widely applied for clinical immunotherapy to fight against a fraction of advanced cancer (Fig. 1). The approved or ongoing clinical trials mAbs that regulate PD-1/PD-L1 pathway were listed in Table 1. Nivolumab was the first mAb against PD-1 approved by FDA for the treatment of unresectable or metastatic melanoma based on its remarkable survival benefits in clinical trials [48]. Subsequently, the second PD-1 antibody, pembrolizumab, was designated for the treatment of advanced melanoma due to its manageable irAEs and significant improvements in prognosis and fewer treatment-related adverse events than chemotherapy [49]. This therapeutic effect was also found in patients with non-small-cell lung cancer (NSCLC), locally advanced or metastatic esophageal cancer and in patients with advanced or metastatic gastric or gastroesophageal junction cancer with microsatellite instability-high (MSI-H) status [50–52]. In addition, PD-1 antibodies include Cemiplimab, Dostarlimab and Toripalimab, whose therapeutic effects have been confirmed in various solid tumors [53–55]. Except for the encouraging performance of PD-1 antibodies, the PD-L1 antibodies have also shown obvious clinical benefits for multilple malignancies. For instance, avelumab, atezolizumab, and durvalumab have been proven beneficial activity and safety for treatment of locally advanced or metastatic urothelial cancer [56–58]. In the context of NSCLC, envafolimab, durvalumab, and sugemalimab were confirmed to achieve obvious improvements in prognosis with limited drug toxicity [59–61]. Nevertheless, it has also been demonstrated that not all patients with solid malignancies can benefit from PD-1/PD-L1 antibodies, and some patients experience hyperprogression, which is a remaining challenge in immunotherapy [62–64]. Therefore, the current research direction of PD-1/PD-L1 is to select individualized treatment plans, find potential benefit groups, combine multiple targeted ICIs, and timely adjust appropriate immunotherapy after immune resistance.

**Table 1:** The approved or ongoing clinical trials mAbs that target immune checkpoints.

mAb name	Another name	Target	IgG isotype	Main types of cancer applicable	Function	Reference	Clinical Trial stage
Nivolumab	OPDIVO	PD-1	IgG4	Unresectable or metastatic melanoma; Non-small-cell lung cancer (NSCLC)	Blocks the interaction between PD-1 and PD-L1 or PD-L2	[48]	FDA approved
Pembrolizumab	KEYTRUDA	PD-1	IgG4	Unresectable or metastatic melanoma; Metastatic squamous NSCLC	Blocks the interaction between PD-1 and PD-L1 or PD-L2	[49]	FDA approved
Cemiplimab	LIBTAYO	PD-1	IgG4	Metastatic or locally advanced cutaneous squamous cell carcinoma (mCSCC or laCSCC); Locally advanced or metastatic basal cell carcinoma (laBCC or mBCC); Locally advanced or metastatic NSCLC	Blocks the interaction between PD-1 and PD-L1 or PD-L2	[53]	FDA approved
Dostarlimab	JEMPERLI	PD-1	IgG4	Mismatch repair defect (dMMR) recurrent or advanced endometrial cancer	Blocks the interaction between PD-1 and PD-L1 or PD-L2	[54]	FDA approved
Toripalimab	LOQTORZI	PD-1	IgG4	Recurrent unresectable or metastatic nasopharyngeal carcinoma (NPC) with disease progression on or after a platinum-containing chemotherapy; First-line treatment of metastatic or with recurrent locally advanced NPC (In combination with Cisplatin and Gemcitabine)	Blocks the interaction between PD-1 and PD-L1 or PD-L2	[55]	FDA approved
Avelumab	BAVENCIO	PD-L1	IgG1	Metastatic Merkel cell carcinoma (MCC)	Blocks the interaction between PD-1 and PD-L1	[56]	FDA approved
Atezolizumab	TECENTRIQ	PD-L1	IgG1	Locally advanced or metastatic urothelial carcinoma	Blocks the interaction between PD-1 and PD-L1	[57]	FDA approved
Durvalumab	IMFINZI	PD-L1	IgG1	Locally advanced or metastatic urothelial carcinoma	Completely blocks the binding of PD-L1 to PD-1 or CD80	[58]	FDA approved
Envafolimab	KN035	PD-L1	IgG1	Microsatellite stable (MSS) locally advanced rectal adenocarcinoma; NSCLC	Inhibits the binding of PD-L1 to PD-1 or CD80	[59]	Phase III study
Sugemalimab	CEJEMLY	PD-L1	IgG4	Unresectable stage III NSCLC (In combination with chemotherapy); Limited-stage small-cell lung cancer (LS-SCLC) who have not progressed following concurrent or sequential chemoradiotherapy; Extranodal NK/T-cell Lymphoma	Inhibits the binding of PD-L1 to PD-1 or CD80	[61]	Phase II/III study
Ipilimumab	YERVOY	CTLA-4	IgG1	Unresectable or metastatic melanoma; Microsatellite instability (MSI-H) or dMMR metastatic colorectal cancer (Phase III study); Metastatic NSCLC (Phase III study)	Blocks the interaction between CTLA-4 and B7	[70]	FDA approved
Tremelimumab	IMJUDO	CTLA-4	IgG2	Unresectable hepatocellular carcinoma (In combination with durvalumab); Metastatic NSCLC (In combination with durvalumab and platinum-based chemotherapy)	Blocks the interaction between CTLA-4 and B7	[76]	FDA approved
Relatlimab	BMS-986016	LAG-3	IgG4	Unresectable or metastatic melanoma (In combination with Nivolumab)	Blocks the immunosuppressive effects of LAG-3	[88]	FDA approved
Sabatolimab	MBG453	TIM-3	IgG4	Intermediate, high or very high risk myelodysplastic syndrome (MDS) as per IPSS-R or Chronic Myelomonocytic Leukemia-2 (CMML-2)	Blocks TIM-3 to restore the anti-tumor activity of T cells; Directly targets leukemia stem cells (LSCs)	[109]	Phase III study
Cobolimab	TSR-022	TIM-3	IgG4	NSCLC	Blocks TIM-3 to restore the anti-tumor activity of T cells	[96]	Phase II/III study

### CTLA-4

**Fig. 2:**
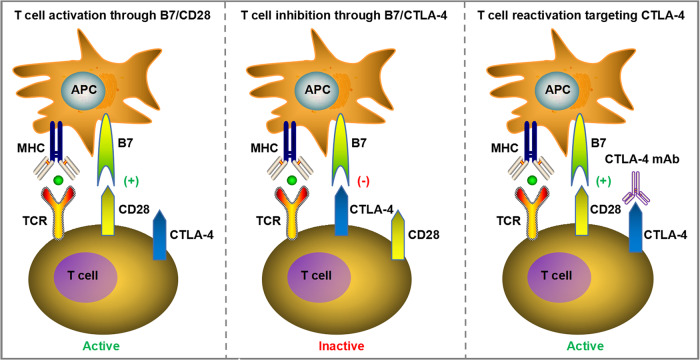
CTLA-4 negative regulates T cell responses through several ways, such as attenuating T cell receptor (TCR) and CD28 signaling by competively binding to B7 with CD28. The application of mAbs to block CTLA-4 can relieve its inhibitory effects on T cells, reactivate T cell proliferation and differentiation into cytotoxic T lymphocytes (CTLs), thereby exerting anti-tumor immune effects.

CTLA-4 (also known as CD152) is another transmembrane receptor that shares the B7 ligand with co-stimulatory receptor CD28. CTLA-4 is mainly expressed on the surface of immunosuppressive T regulatory cells (Tregs) and only on the activated conventional T lymphocytes [65]. In view of the homology of CTLA-4 and CD28, they both bind to the same B7 ligands CD80 (B7-1) and CD86 (B7-2), but CTLA-4 has a higher affinity than CD28 [66]. CTLA-4 mainly plays a role in the inhibitory modulation of T cell activation and induction of T cell immune tolerance. It has been verified that CTLA-4 can impede T cell proliferation and differentiation, induce cell cycle arrest, and reduce IL-2 production to exert the inhibitory function in T cell modulation [67]. CTLA-4 has been reported to mediate negative regulation of T cell responses through several ways, such as attenuating the T cell receptor (TCR) and CD28 signaling by competively binding to B7 with CD28 or recruiting phosphatases to the intracellular domain of CTLA-4, and another mechanism is the induction of Treg development and function. Additionally, CTLA-4 was found to decrease CD80 and CD86 expression on antigen presenting cells (APCs) or remove them from APCs by transendocytosis to hinder the participation of CD28 in T cell activation process [68, 69]. Therefore, the application of monoclonal antibody to block CTLA-4 can relieve its inhibitory effects on T cells, reactivate T cell proliferation and differentiation into cytotoxic T lymphocytes (CTLs), thereby exerting anti-tumor immune effects (Fig. 2).

Currently, the mainstream monoclonal antibodies targeting CTLA-4 are ipilimumab and tremelmumab (Table 1). Ipilimumab is a humanized IgG1 monoclonal antibody that binds to CTLA-4 in the B7 interaction domain, mediating steric hindration to block the binding of CTLA-4 to B7, and is the first CTLA-4 antibody drug used in advanced melanoma. A number of clinical studies have shown that Ipilimumab has a high rate of disease control and benifits for improving prognosis [70–72]. Moreover, most existing studies have focused on the combination therapy of ICIs. In NSCLC patients, compared with neoadjuvant nivolumab, the combination therapy of nivolumab and ipilimumab can significantly improve the pathological complete response rates, tumor immune infiltrates and immune memory effects [73]. Another multicenter, randomized Phase III clinical trial (CheckMate 9LA) also confirmed the feasibility of this treatment [74, 75]. Tremelimumab is another humanized IgG2 monoclonal antibody against CTLA-4, which binds to CTLA-4 and blocks its interaction with ligands CD80 and CD86, thereby enhancing T cell response to tumor cells [76]. In October 2022, the FDA approved tremelimumab in combination with durvalumab for the treatment of adult patients with unresectable hepatocellular carcinoma (uHCC) based on data from HIMALAYA’s Phase III clinical trial [77–79]. In November 2022, based on the Phase III POSEIDON clinical trial, tremelimumab, durvalumab, and platinum-containing chemotherapy combinations are also approved by the FDA for the treatment of metastatic NSCLC without EGFR-sensitive mutations and ALK rearrangements [77, 80]. However, the overall rate of adverse events in patients receiving ICIs combination therapy is higher than in the monotherapy group, and not every specific cancer patient will benefit from it [81]. Therefore, future studies may be urgently needed to improve the clinical outcomes of such combination therapies and reduce the incidence of adverse events.

### LAG-3

**Fig. 3:**
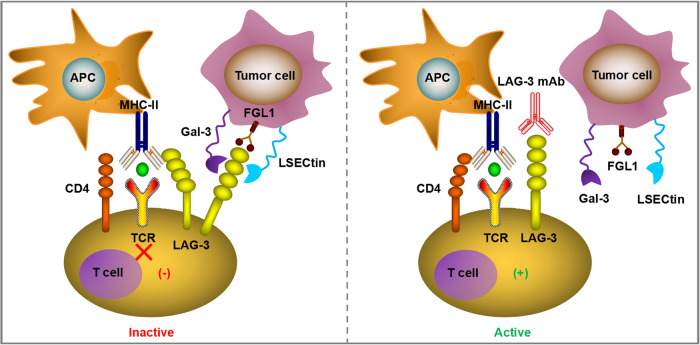
The typical ligand of LAG-3 is MHC class II (MHC-II), which interacts stably with LAG-3 through the D1 domain and has a significantly higher affinity than CD4. The high-affinity LAG-3Ig/MHC-II binding competes with CD4/MHC-II interaction to mediate the suppressed T cell proliferation and inhibitory immune response. The second ligand of LAG-3 is Gal-3, which has been found to be expressed in multiple cancer cells and activated T cells. Gal-3 can interact with LAG-3 and this binding is essential for the inhibitory cytotoxicity of CD8 + T lymphocytes. Other newly identified LAG-3 functional ligands include FGL1 and LSECtin. The blockade of LAG-3 with mAbs can relieve the T cell inhibitory immune response triggered by the interaction between LAG-3 and its ligands.

LAG-3 (also known as CD223), the third immune checkpoint applied in clinical practice after CTLA-4 and PD-1/PD-L1, is an inhibitory transmembrane receptor found on the surface of effector T cells, regulatory T cells, natural killer cells, activated B cells, and plasmacytoid dendritic cells [82]. The LAG-3 gene includes eight exons and its encoded protein can be divided into extracellular, transmembrane, and intracellular domains, which is closely related to CD4 [83]. The typical ligand of LAG-3 is MHC Class II (MHC-II), which interacts stably with LAG-3 through the D1 domain. It was initially thought that high-affinity LAG-3/MHC-II binding competes with CD4/MHC-II interactions to inhibit the proliferation, activation, cytotoxicity and cytokine production of CD4+ and CD8 + T lymphocytes [84, 85]. However, whether the LAG-3/MHC-II interaction alone is responsible for LAG-3 immunosuppressive signaling remains controversial, given the recent discovery of other ligands, including galactin-3 (Gal-3), fibrinin-as-protein 1 (FGL1), and LSECtin. Gal-3 is expressed in a variety of cancer cells and activated T cells, and it can interact with LAG-3 to inhibit cytotoxicity of CD8 + T lymphocytes [84–86]. Another newly discovered LAG-3 functional ligand, FGL1, is a member of the liver-secreted fibrinogen family of proteins and is highly expressed in a variety of tumor cells, such as melanoma, lung cancer, and colorectal cancer cells [87]. The binding of FGL1 and LAG-3 inhibits antigen-specific T cell immune response, and blocking the interaction between FGL1 and LAG-3 can reactivate the anti-tumor immune ability of T cells [87]. Interestingly, upregulation of FGL1 expression may also be associated with poor prognosis and anti-PD-1 resistance [87]. Thus, blocking LAG-3 with monoclonal antibodies mitigated the T-cell suppressive immune response triggered by the interaction between LAG-3 and its ligand (Fig. 3).

The efficacy of LAG-3 mAbs is currently being studied in a variety of tumors. Relatlimab is a first-of-its-kind human IgG4 anti-LAG-3 antibody that binds to LAG-3 and restores T cell function [88]. Meanwhile, studies have found that LAG-3 and PD-1 were widely co-expressed on CD4+ and CD8 + T cells, especially on tumor-infiltrating T cells [89, 90]. LAG-3 in combination with the typical inhibitory immune checkpoint PD-1/PD-L1 can co-mediate immune homeostasis, eliminate autoimmune diseases, and enhance tumor-induced tolerance [90, 91]. The FDA has approved Relatlimab in combination with PD-1 mAb Nivolumab for the treatment of unresectable or metastatic melanoma [92, 93] (Table 1). In addition, LAG-3 mAb and PD-1 mAb have also been shown to have significant synergistic effects in colorectal cancer [94, 95]. In spite of the potential application value of LAG-3 in cancer immunotherapy, its functional mechanisms remains obscure. It is important to have a deep understanding of the action mechanism of LAG-3 for identifying its unknown effects and developing better therapeutic methods to block or replicate LAG-3 function, as well as searching for suitable biomarkers and selecting suitable patients to maximize the effect of LAG-3 mAb.

### TIM-3

**Fig. 4:**
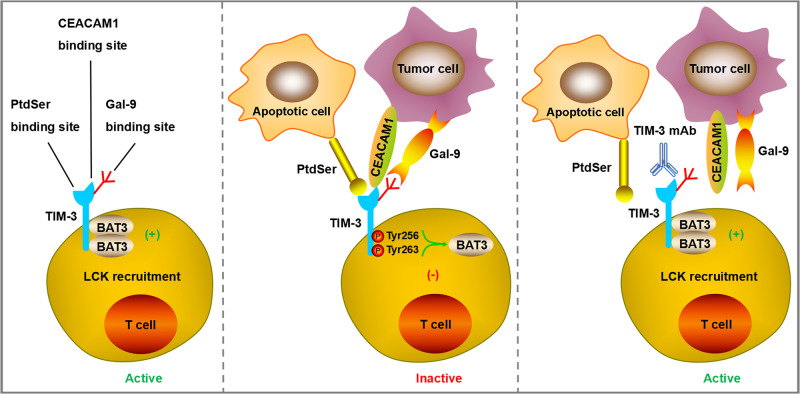
TIM-3 has multiple ligands, such as Gal-9, CEACAM1, PtdSer, and HMGB1, and each of them binds to different regions of the extracellular IgV domain. When TIM-3 is not bound to the ligand, it interacts with BAT3 and maintains T cell activation through Tyrosine kinase LCK recruitment. Once TIM-3 binds to the ligand, BAT3 is released from TIM-3 and Fyn binds to it, allowing TIM-3 to exert its inhibitory function. Most mAbs targeting TIM-3 hinder the binding of ligands to TIM-3, thereby maintaining the binding state between TIM-3 and BAT3.

TIM-3, also known as hepatitis A virus cell receptor 2 (HAVCR2) or CD366, is another promising inhibitory receptor among numerous emerging immune checkpoints and has achieved initial anti-tumor effects in clinical trials of its monotherapy or combination with anti PD-1/PD-L1 drugs [96]. TIM-3 as an important member of the TIM family protein, it contains a C-terminal cytoplasmic tail, a single transmembrane domain signal peptides, a mucinlike domain and an extracellular N-terminal variable immunoglobulin (IgV) domain [97]. TIM-3 has been reported to have multiple ligands, such as galectin-9 (Gal-9), carcinoembryonic antigen related cell adhesion molecule 1 (CEACAM1), phosphatidylserine (PtdSer) and high mobility group box 1 (HMGB1), and each of them binds to different regions of the extracellular IgV domain [98]. The ligand Gal-9 binding to TIM-3 was found to induce apoptosis of Th1 effector cells and reduce IFN-γ production [99]. The ligand CEACAM1 binding to TIM-3 plays a crucial role in regulating cell-mediated autoimmunity and anti-tumor immunity [100]. The ligand PtdSer binding to TIM-3 promotes clearance of apoptotic vesicles to maintain immune tolerance [101]. TIM-3 is preferentially expressed on CD4 + T cell subsets (Th1/Th17), CD8 + T cell subsets (Tc1) and regulatory T cells (Tregs) to generate inhibitory signals, resulting in inhibition of T cell activation and proliferation, reducing the production of cytokines that positively regulate immune responses, and playing a negative regulatory role in the process of anti-tumor immunity [102]. Additionally, it is demonstrated that TIM-3 is also expressed on the surface of DCs, which can mediate the phagocytosis of apoptotic cells and the cross-presentation of antigens [103]. Researchers found that TIM-3 on DCs but not on CD4+ or CD8 + T cells has singular function to restrain anti-tumor immunity through impeding the maintenance of effector T cells and stem-like CD8 + T cells, resulting in inhibition of protective immune effects and reduction of NLRP3 inflammasome activation [104]. Except for DCs, TIM-3 was also detected on the surface of other innate immune cells, such as NK cells, monocytes and macrophages [105]. When TIM-3 is not bound to the ligand, it interacts with HLA-B associated transcription factor 3 (BAT3) and maintains T cell activation through Tyrosine kinase LCK recruitment. Once TIM-3 binds to the ligand, BAT3 is released from TIM-3 and Fyn binds to it, allowing TIM-3 to exert its inhibitory function [106, 107]. Most antibodies targeting TIM-3 hinder the binding of ligands to TIM-3, thereby maintaining the binding state between TIM-3 and BAT3 (Fig. 4). However, the affinity and function of TIM-3 binding to its ligand have not yet been fully elucidated.

TIM-3 can serve as a biomarker for T cell exhaustion, and targeting TIM-3 with monoclonal antibodies can restore T cell vitality, allowing it to fight against tumor cells [108] (Table 1). Sabatolimab (MBG453) is a humanized representative anti-TIM-3 IgG4 antibody developed by Novartis that directly targets the TIM-3 receptor with immuno-myeloid activity to fight against myeloid cell neoplasms [109]. As a single agent, TIM-3 antibody has no substantial clinical benefits, but when combined with other checkpoint inhibitors, such as PD-1 antibody, it can elicit a stronger immune response. A prospective clinical study involving 219 cancer patients showed that MBG453 in combination with PD-1 mAb spartalizumab presented beneficial clinical activity and was also well tolerated with no dose-limiting toxicities observed [110]. Researchers also found that T cells co-expressing TIM-3/PD-1 showed more severe exhausted phenotype, deprived of proliferative viability and unable to produce IL-2, TNF, and IFN-γ. The study further confirmed that combined targeting TIM-3 and PD-1 showed stronger effects to restore anti-tumor immunne response and restrict tumor growth than blocking a single target alone [111]. Cobolimab (TSR-022) is another selective TIM-3 targeting monoclonal antibody developed by Tesaro that can activate immune cell function. When used in combination with anti PD-1 drugs such as dostarlimab, it can elicit strong anti-tumor activity without severe adverse events [96]. Currently, a phase 2 clinical trial (NCT03680508) is addressing the safety and efficacy of cobolimab in combination with dostarlimab in the context of liver cancer [96]. Another phase 2/3 clinical trial (NCT04655976) is evaluating its combination application with docetaxel and dostarlimab in NSCLC patients with prior progressive disease to anti-PD-1/PD-L1 drugs [96]. Although TIM-3 is currently one of the most studied immune checkpoints, there are no commercially available drugs nationally and internationally. This may be due to the complexity of the mechanism and the unclear beneficiaries. Therefore, the study of the combined application of anti TIM-3 and anti PD-1/PD-L1 drugs may ameliorate the resistance of PD-1/PD-L1, save T cell failure, and improve the prognosis of patients.

### Other immune checkpoints

Except for the above major well-known immune checkpoints, there are several other emerging molecules on the investigation for negative immune regulation with immunotherapy targeting potential. For example, T cell immunoglobulin and ITIM domain (TIGIT), also known as WUCAM, Vstm3, VSIG9, is an inhibitory immune checkpoint molecule belonging to the poliovirus receptor (PVR)/Nectin family and is expressed on CD4 + T cells, CD8 + T cells, Treg cells, and NK cells [112, 113]. TIGIT can bind to its ligands CD155 and CD112 to inhibit innate and adaptive immune responses via different mechanisms [114]. There are currently various mAbs targeting TIGIT on the investigation either as a monotherapy or combined therapy with PD-1/PD-L1 antibodies. Combination therapy of TIGIT mAb tiragolumab and PD-L1 mAb atezolizumab has been reported to enhance overall response rate in the context of metastatic NSCLC with PD-L1 positive expression [115].

V domain Ig inhibitory factor activated by T cells (VISTA, also known as VSIR, Gi24, Dies-1, PD-1H, B7-H5, SISP1, or DD1α), is another inhibitory immune checkpoint that is predominantly expressed in myeloid cells (monocytes, macrophages, neutrophils and DCs), but also found in T lymphocytes and NK cells [116, 117]. VSIG-3 (also known as BTIgSF, IgSF11 or Igsf13) is a VISTA ligand and this interaction inhibits T cell responses [117]. The blockade of VISTA enhances T cell activation and the upregulates the production of many inflammatory cytokines, such as IFN-γ, IL-2, IL-17, CCL-3, CCL-5, and CXCL-11 [118]. Furthermore, VISTA has the potential to reinforce the function of myeloid-derived immune suppressor cells, maintaining the survival of Tregs, restraining the antigen presentation ability of APCs [119]. However, the exact regulatory functions and mechanisms of VISTA in tumor immunity remain to be fully elucidated. Other suppressive immune checkpoints that are being explored as promising immunotherapeutic targets include signal regulatory protein α (SIRPα), B and T lymphocyte attenuator (BTLA), leukocyte immunoglobulin-like receptor subfamily B member 4 (LILRB4), and sialic acid-binding immunoglobulin-like lectin 7 (Siglec-7).

**Fig. 5:**
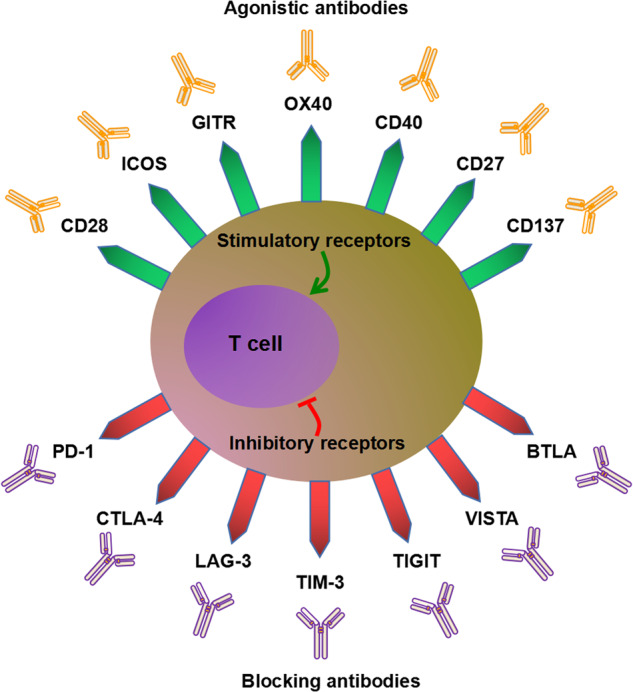
The agonistic antibodies targeting stimulatory receptors and the blocking antibodies targeting inhibitory receptors applied in anti-tumor immunity. The common stimulatory and inhibitory immune checkpoint receptors with positive and negative regulatory effects on anti-tumor immunity, and the blocking antibodies targeting inhibitory receptors, and the agonistic antibodies targeting stimulatory receptors.

Apart from these inhibitory immune checkpoints, some stimulant checkpoint molecules with the potential of positive immunoregulation have also been considered for anti-tumor immunotherapy. CD28 and immune co-stimulator (ICOS) belonging to B7-CD28 superfamily members are critical activating receptors expressed on T cells and participate in the survival, expansion, and function of effector T cells and Tregs [120–122]. The ICOS agonist mAb vopratelimab has been reported to present a beneficial safety profile alone and in combination with nivolumab, but the clinical activity was limited and only effective in a portion of patients with specific pharmacodynamic biomarkers of vopratelimab [123]. Additionally, checkpoint molecules belonging to the tumor necrosis factor receptor superfamily (TNFR-SF), such as glucocorticoid-induced TNFR-related gene (GITR/TNFRSF18/CD357), TNFRSF4 (OX40/CD134), TNFRSF5 (CD40), TNFRSF7 (CD27), and TNFRSF9 (CD137/4-1BB), have also been defined as stimulatory immune checkpoints and under evaluation as potential targets for intervention in the field of cancer immunotherapy [124]. We have summarized the common stimulatory and inhibitory immune checkpoint receptors with positive and negative regulatory effects on anti-tumor immunity in Fig. 5. These newly discovered immune checkpoints provide ideas to address primary and acquired resistance to PD-1/PD-L1 therapy, and may even replace existing PD-1/PD-L1 therapy in the future. It is also possible to develop new ICIs drugs by focusing on the immune checkpoints of the remaining cells in the immune microenvironment, including NK cells, DCs and etc, thereby extending the survival time of patients and improving their prognosis.

### CircRNAs are involved in the hallmarks of cancer

For a long time, circRNAs have been mistakenly thought to be by-products of mRNA splicing, with no specific function. Until 2012, this dark matter was unexpectedly found to be ubiquitously expressed in eukaryotic genomes, which revealed fundamental issues respecting the promising biological function of circRNAs [125]. A deep sequencing study of 2000+ clinical tissue samples and cell lines from more than 10 cancer types revealed that circRNAs with stable structures may serve as the cancer biomarkers in blood or urine [126]. As research on circRNAs and cancer hallmarks in tumors has grown significantly over the past decade, it is impossible for this review to cover every reported study. For each hallmark, we will present the most representative examples. We have summarized the targets and mechanisms by which selected circRNAs are involved in the hallmarks of cancer in Table 2.

**Table 2:** CircRNAs involved in the hallmarks of cancer.

circRNA name	circBase ID	Parental gene	Target	Cancer type	Function	Mechanism	Reference
circFAM120A	-	FAM120A	FAM120A	-	Inhibits proliferation	Inhibits the binding of IGF2BP2 and FAM120A to promote FAM120A translation	[131]
circVAMP3	-	VAMP3	c-Myc	Hepatocellular carcinoma	Inhibits proliferation and metastasis	Promotes the phase separation of CAPRIN1 protein to form stress granules in cells to prevent the translation of c-Myc mRNA	[130]
circPCNX	hsa_circ_0032434	PCNX	p21	Cervical carcinoma	Inhibits proliferation	Binds to AUF1 and hinder the binding of p21 to AUF1 to enhance the mRNA stability and protein production of p21	[132]
circTP63	hsa_circ_0068515	TP63	FOXM1, CENPA, CENPB	Lung squamous cell carcinoma	Facilitates cell cycle progression and promotes cell proliferation	Binds to miR-873-3p to upregulate FOXM1 expression, and then upregulates downstream molecules CENPA and CENPB	[128]
circPINTexon2	-	LINC-PINT	CPEB1, SOX-2, c-Myc, etc	Glioblastoma	Inhibits proliferation	Encodes protein PINT87aa which interacts with PAF1c and suppresses the transcriptional elongation of multiple oncogenes	[133]
BCRC-3	-	PSMD1	p27	Bladder cancer	Inhibits proliferation	Acts as a miR-182-5p sponge to upregulate p27 expression	[134]
circPVT1	-	PVT1	E2F2	Gastric cancer	Promotes proliferation	Acts as a sponge for miR-125 family members to upregulate E2F2 expression	[129]
circHIPK3	-	HIPK3	-	-	Inhibits proliferation	Sponges with miR-124 and inhibits its activity	[135]
circFoxo3	hsa_circ_0006404	Foxo3	-	Bladder cancer, gastric cancer, glioma, acute lymphocytic leukemia, etc	Promotes or inhibits apoptosis	Acts as a sponge for various miRNAs	[212]
circ-MAPK4	has_circ_0047688	MAPK4	p38/MAPK	Glioma	Inhibits apoptosis	Functions as miR-125a-3p sponge to regulate p38/MAPK signaling pathway	[195]
circ-TTBK2	-	TTBK2	HNF1β, Derlin-1	Glioma	Inhibits apoptosis	Acts as miR-217 sponge to target HNF1β/Derlin-1 pathway	[213]
circATG4B	hsa_circ_0007159	ATG4B	-	Colorectal cancer	Promotes autophagy	Encodes a novel protein, circATG4B-222aa, which competitively binds to TMED10 and prevent the interaction of TMED10 and ATG4B	[216]
circATG7	has_circ_0064288	ATG7	ATG7	Pancreatic cancer	Facilitates cell autophagy, proliferation and invasion	Functions as miR-766-5p sponge to regulate miR-766-5p/ATG7 and HUR/ATG7 axis	[217]
circCDYL	hsa_circ_0008285	CDYL	ATG7, ULK1	Breast cancer	Augments cell autophagy	Functions as miR-1275 sponge to regulate the miR-1275-ATG7/ULK1 axis	[218]
circPIBF1	-	PIBF1	SOD2	Lung adenocarcinoma	Inhibits cell pyroptosis	Binds to Nrf2, which further promotes SOD2 expression and recruits EP300 to promote H3K27ac modification of SOD2, thus modulating the Nrf2/Keap-1 pathway	[220]
circ7312	hsa_circ_0007312	-	MAPK1	Lung adenocarcinoma	Inhibits cell pyroptosis and apoptosis	Functions as miR-766-5p sponge to regulate miR-764/MAPK1 axis	[221]
circNEIL3	hsa_circ_0001460	NEIL3	PIF1	Lung adenocarcinoma	Mediates radiation-induced pyroptosis	Functions as miR-1184 sponge to upregulate PIF1 expression	[222]
circPUM1	hsa_circ_0011240	PUM1	-	ESCC	Inhibits cell pyroptosis	Acts as a scaffold for the interaction between UQCRC1 and UQCRC2	[223]
circSCN8A	hsa_circ_0026337	SCN8A	ACSL4	NSCLC	Induces cell ferroptosis	Serves as a miR-1290 sponge to activate ACSL4 expression	[227]
circST6GALNAC6	-	ST6GALNAC6	P38 MAPK	Bladder cancer	Facilitates cell ferroptosis	Binds to HSPB1 and blocks erastin-induced phosphorylation site of HSPB1, protectively in response to ferroptosis stress (Ser-15 site), thus activating the P38 MAPK pathway	[228]
circRNA_101093	-	-	FABP3	Lung adenocarcinoma	Desensitizes lung adenocarcinoma cells to ferroptosis	Binds to FABP3 and increases its expression	[229]
circ-TPGS2	-	TPGS2	TRAF6	Breast cancer	Promotes tumor-associated inflammation	Serves as a miR-7 sponge to increase TRAF6 expression, contributing to p65 phosphorylation and nuclear translocation, ultimately inducing the activation of NF-κB signaling pathway and dysregulation of TME	[234]
hsa_circRNA_002178	-	-	COL1A1	Breast cancer	Activates inflammation	Serves as a miR-328-3p sponge to upregulate COL1A1	[235]
mmu_circ_0001109	mmu_circ_0001109	PRKAR2A	-	Colitis-associated colorectal carcinoma (CAC)	Promotes the transition of inflammation to carcinoma	Activates the Wnt/β-catenin signaling pathway	[236]
circNEIL3	hsa_circ_0001460	NEIL3	PIF1	Lung adenocarcinoma	Activates AIM2 inflammasome	Functions as miR-1184 sponge to upregulate PIF1 expression	[222]
circRNA-MYLK	-	MYLK	VEGFA/ VEGFR2	Bladder cancer	Promotes angiogenesis and metastasis	Functions as miR-29a sponge to activate VEGFA/VEGFR2 and downstream Ras/ERK pathway	[241]
circ3823	hsa_circ_0001821	PVT1	Tcf7	Colorectal cancer	Promotes tumor growth, metastasis and angiogenesis	Functions as miR-30c-5p sponge to upregulate Tcf7 expression	[242]
circHIPK3	hsa_circ_0000284	HIPK3	HPSE	Bladder cancer	Inhibits angiogenesis	Functions as miR-558 sponge to negatively regulate the expression of HPSE	[243]
circ-ASH2L	-	ASH2L	Notch 1	Pancreatic ductal adenocarcinoma	Promotes angiogenesis	Acts as a sponge for miR-34a to upregulate Notch 1 expression	[244]
circ-CCAC1	-	ERBB2	YY1	Cholangiocarcinoma	Induces angiogenesis	Functions as miR-514a-5p sponge to upregulate YY1	[245]
circSHKBP1	hsa_circ_0000936	SHKBP1	HUR, VEGF	Gastric cacner	Promotes angiogenesis	Sponges miR-582-3p to activate the HUR/VEGF signaling axis, and directly interact with HSP90 to disrupt the binding of HSP90 and STUB1, further restraining the ubiquitination and degradation of HSP90	[246]
circPTCH1	hsa_circ_0139402	PTCH1	MMP14	Renal cell carcinoma	Facilitates invasion and metastasis	Sponges miR-485-5p to upregulate MMP14 expression	[136]
circNHSL1	hsa_circ_0006835	NHSL1	SIX1	Gastric cacner	Enhances invasion and metastasis	Sponges miR-1306-3p to upregulate SIX1/vimentin axis	[137]
circHIPK3	hsa_circ_0000284	HIPK3	FAK, IGF1R, EGFR, YY1; HPSE; IGF2BP3	Colorectal cancer, bladder cancer, glioma	Promotes or inhibits invasion and metastasis	Sponges miR-7 to upregulate the expression of FAK, IGF1R, EGFR, YY1; sponges miR-558 to suppress HPSE expression; sponges miR-654 to promote IGF2BP3 expression	[135, 139, 140, 162, 243]
circ0001361	hsa_circ_0001361	FNDC3B	MMP9	Bladder cancer	Promotes invasion and metastasis	Sponges miR-491-5p to upregulate MMP9 expression	[141]
circPTEN1	hsa_circ_0002232	PTEN1	TGF-β, Smad	Colorectal cancer	Inhibits invasion and metastasis	Binds the MH2 domain of Smad4 to block its interaction with p-Smad2/3, further disrupting TGF-β/Smad signaling pathway	[144]
circLONP2	hsa_circ_0008558	LONP2	-	Colorectal carcinoma	Promotes invasion and metastasis	Promotes the processing, maturation and exosomal dissemination processes of miR-17-5p	[142]
circANKS1B	hsa_circ_0007294	ANKS1B	TGF-β1	Breast cancer	Promotes invasion and metastasis	Sponges miR-148a-3p and miR-152-3p to enhance the expression of transcription factor USF1, which transcriptionally upregulates TGF-β1, thus activating TGF-β1/Smad signaling pathway to induce EMT	[143]

### CircRNAs involvement in proliferation, migration, invasion and metastasis

Cancer cells have been found to continuously expand and grow by releasing a large number of growth-related signaling factors or expressing excessive growth signaling receptors [127]. A large number of studies have shown that circRNAs can affect the function of tumor proliferation, migration, invasion and metastasis. CircTP63 competitively bind to miR-873-3p to upregulate the expression of FOXM1, which further increase the level of downstream molecules CENPA and CENPB, thereby facilitating cell proliferation in lung squamous cell carcinoma [128]. Similarly, circPVT1 was found to promote cell proliferation in gastric cancer [129]. In addition, there are also various circRNAs that display an inhibitory role in tumor proliferation. CircVAMP3 inhibited the proliferation and migration of hepatocellular carcinoma by promoting the phase separation of CAPRIN1 protein to form stress granules in cells to prevent the translation of c-Myc mRNA [130]. CircFAM120A [131], circPCNX [132], circPINTexon2 [133], BCRC-3 [134] and circHIPK3 [135] have been shown to inhibit tumor proliferation in hepatocellular carcinoma, cervical carcinoma, glioblastoma, bladder cancer and esophageal squamous cell carcinoma, respectively.

Migration, invasion and metastasis are considered to be a complex cascade of biological events in cancer, involved in the spread and dissemination of cancer cells from the primary site to local site and foreign tissues, and tumors tend to exhibit the same trends in all three functions. In recent years, accumulated circRNAs have been reported to play important roles in cancer migration, invasion and metastasis through diverse mechanisms [34]. For instance, circPTCH1 (hsa_circ_0139402) was reported to facilitate migration, invasion and metastasis in renal cell carcinoma (RCC) through modulating miR-485-5p/MMP14 signaling pathway [136]. CircNHSL1 (hsa_circ_0006835) can act as a competitive endogenous RNA (ceRNA) to sponge miR-1306-3p to regulate SIX1/vimentin pathway, leading to enhanced migration, invasion and metastasis ability of gastric cancer cells in vitro and in vivo [137]. CircHIPK3 (hsa_circ_0000284) [138–140], hsa_circ_0001361 (circ0001361) [141], circLONP2 (hsa_circ_0008558) [142] and circANKS1B (hsa_circ_0007294) [143] can promote tumor migration, invasion and metastasis through the sponge mechanism. However, there are also circRNAs can inhibit tumor migration, invasion and metastasis. For example, circPTEN1 (hsa_circ_0002232) could attenuate invasion and metastasis of colorectal cancer through binding the MH2 domain of Smad4 to block its interaction with p-Smad2/3, further disrupting TGF-β/Smad signaling pathway [144].

**Fig. 6:**
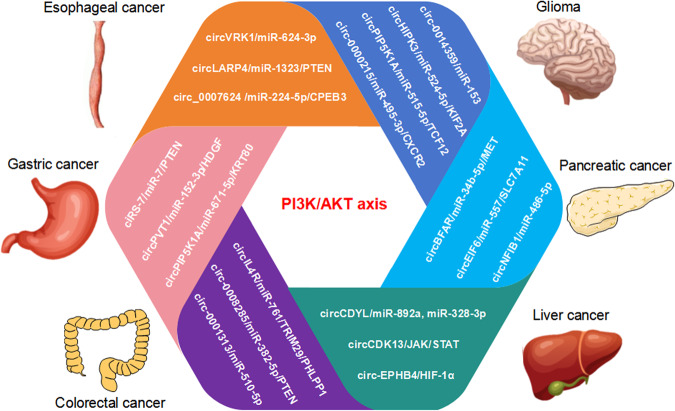
CircRNAs target PI3K/AKT axis to regulate tumor cell proliferation and metastasis. The circRNAs target PI3K/AKT axis to regulate cell proliferation and metastasis in the context of various cancers, such as circVRK1/miR-624-3p/PTEN, circLARP4/miR-1323/PTEN, and circ_0007624/miR-224-5p/CPEB3 pathway targeting PI3K/AKT axis in esophageal cancer; ciRS-7/miR-7/PTEN, circPVT1/miR-152-3p/HDGF, and circPIP5K1A/miR-671-5p/KRT80 pathway targeting PI3K/AKT axis in gastric cancer; circIL4R/miR-761/TRIM29/PHLPP1, circ_0008285/miR-382-5p/PTEN, and circ-0001313/miRNA-510-5p/AKT2 pathway targeting PI3K/AKT axis in colorectal cancer; circCDYL/miR-892a and miR-328-3p, circCDK13/JAK/STAT, and circEPHB4/HIF-1ɑ pathway targeting PI3K/AKT axis in liver cancer; circBFAR/miR-34b-5p/MET, circEIF6/miR-557/SLC7A11, and circNFIB1/miR-486-5p/PIK3R1/VEGF-C pathway targeting PI3K/AKT axis in pancreatic cancer; circ0014359/miR-153, circHIPK3/miR-524-5p/KIF2A, circPIP5K1A/miR-515-5p/TCF12, and circ0000215/miR-495-3p/CXCR2 pathway targeting PI3K/AKT axis in glioma.

In terms of the above mentioned effects of circRNAs on biological functions in various tumors by different mechanisms, we systematically summarized the signaling pathways involved in circRNAs regulating tumor proliferation and metastasis as follows. The PI3K/AKT signaling pathway is an intracellular signaling pathway that regulates various cellular functions, such as metabolism, proliferation, cell survival, growth, and angiogenesis (Fig. 6). In esophageal cancer, circVRK1/miR-624-3p/PTEN, circLARP4/miR-1323/PTEN, and circ_0007624/miR-224-5p/CPEB3 axis were confirmed to regulate PI3K/AKT signaling pathway participating in cell proliferation [145–148]. In gastric cancer, ciRS-7/miR-7/PTEN, circPVT1/miR-152-3p/HDGF, and circPIP5K1A/miR-671-5p/KRT80 axis were reported to involve in cell proliferation [149–151]. In colorectal cancer, circIL4R, circ_0008285, and circ-0001313 have been verified to modulate proliferation phenotype [152–154]. CircCDYL, circCDK13 and circEPHB4 were verified to involve in PI3K/AKT signal regulation to alter hepatocellular carcinoma proliferation ability [155–157]. CircBFAR/miR-34b-5p/MET, circEIF6/miR-557/SLC7A11, and circNFIB1/miR-486-5p/PIK3R1/VEGF-C axis have been reported to be a pancreatic cancer proliferation hallmarker [158–160]. In glioma, circ0014359, circHIPK3, circPIP5K1A, and circ0000215 were confirmed to regulate PI3K/AKT axis involving in cell proliferation [161–164].

**Fig. 7:**
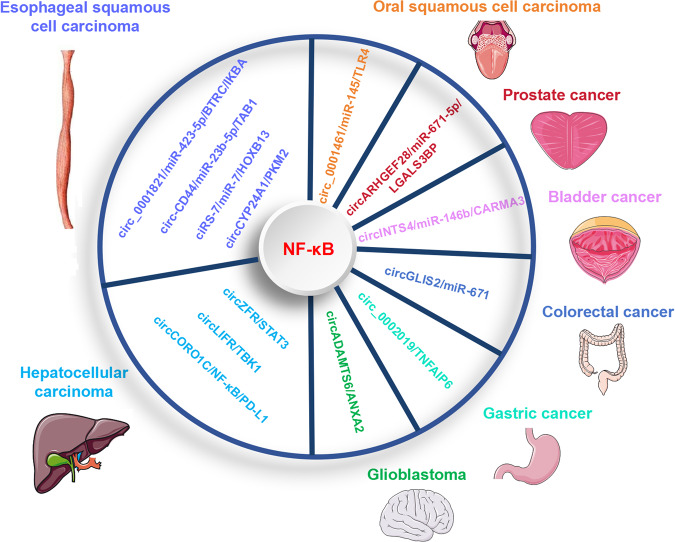
CircRNAs target NF-κB signaling to regulate tumor cell proliferation. The circRNAs target NF-κB signaling to regulate cell proliferation in the context of various cancers, such as circ_0001821/miR-423-5p/BTRC/IKBA, circ-CD44/miR-23b-5p/TAB1, ciRS-7/miR-7/HOXB13, circCYP24A1/PKM2 axis to regulate NF-κB signal in ESCC; circZFR/STAT3/NF-κB, circLIFR/TBK1/NF-κB, circCORO1C/NF-κB/PD-L1 in hepatocellular carcinoma; circADAMTS6/ANXA2/NF-κB in glioblastoma; circ_0002019/TNFAIP6/NF-κB in gastric cancer; circGLIS2/miR-671/NF-κB in colorectal cancer; circINTS4/miR-146b/CARMA3/NF-κB in bladder cancer. circARHGEF28/miR-671-5p/LGALS3BP/NF-κB in prostate cancer; circ_0001461/miR-145/TLR4/NF-κB axis in oral squamous cell carcinoma.

Additionally, the nuclear factor-kappaB (NF-κB) signaling is also considered as a proliferation related pathway in cancer, and multiple circRNAs, such as circ_0001821, hsa_circ_0021727 (circ-CD44), ciRS-7, circCYP24A1, circZFR, circLIFR, circCORO1C, circADAMTS6, circ_0002019, circGLIS2, circINTS4, circARHGEF28, circ_0001461, have been reported to regulate cell proliferation through modulation of NF-κB signaling axis in different tumors [165] (Fig. 7). In the settings of esophageal squamous cell carcinoma (ESCC), circ_0001821 acts as a miR-423-5p sponge to upregulate BTRC expression, thus inducing IKBA ubiquitination to affect NF-κB signaling and cell proliferation [166]; hsa_circ_0021727 (circ-CD44) promotes proliferation by sponging miR-23b-5p to upregulate the TAB1/NF-κB pathway [167]; ciRS-7 promotes ESCC growth and metastasis through miR-7/HOXB13 induced NF-κB pathway [168]; circCYP24A1 facilitates ESCC progression by interacting with PKM2 to regulate NF-κB signaling [169]. Under the context of hepatocellular carcinoma, circZFR promotes hepatocellular carcinoma development by inhibiting the STAT3/NF-κB pathway [170]; circLIFR promotes cell proliferation by interacting with TANK binding kinase 1 (TBK1), which is a serine/threonine kinase that regulates NF-κB pathway [171]; circCORO1C promotes hepatocellular carcinoma proliferation and metastasis via upregulating NF-κB pathway induced PD-L1 expression [172]. During glioblastoma progression, circADAMTS6 facilitates cell proliferation and tumor growth through regulation of ANXA2/NF-κB pathway in a TDP43-dependent way [173]. In gastric cancer, circ_0002019 enhances cell proliferation, migration, and invasion through TNFAIP6/NF-κB signaling [174]. In colorectal cancer, circGLIS2 promotes cell proliferation by functioning as a miR-671 sponge to activate NF-κB signaling pathway [175]. In bladder cancer, circINTS4 facilitates proliferation and tumorigenesis by activating the NF-kB signaling via miR-146b/CARMA3 axis [176]. In prostate cancer, circARHGEF28 inhibits cell proliferation by regulating NF-κB axis through miR-671-5p/LGALS3BP pathway [177]. During oral squamous cell carcinoma progression, circ_0001461 promotes cell proliferation and tumor growth through sponging miR-145 to modulate TLR4/NF-κB signaling axis [178].

**Fig. 8:**
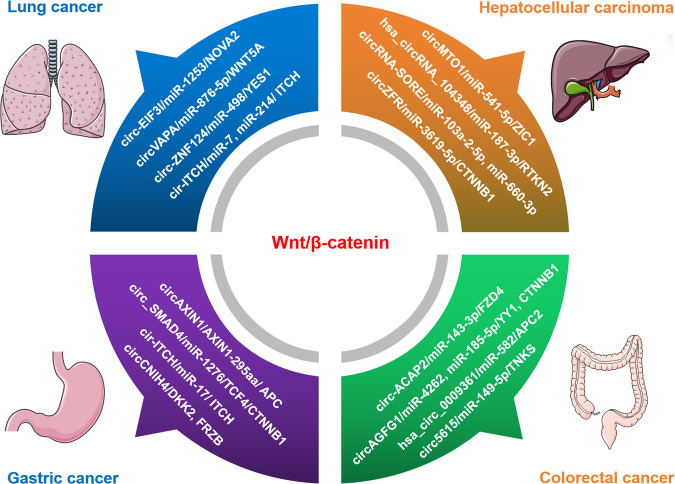
The circRNAs involving in Wnt/β-catenin signaling to regulate cell proliferation in the context of various cancers. In lung cancer, circ-EIF3I/miR-1253/NOVA2, circVAPA/miR-876-5p/WNT5A, circ-ZNF124/miR-498/YES1, and cir-ITCH/miR-7, miR-214/ ITCH pathway have been confirmed to affect the proliferation phenotype by targeting Wnt/β-catenin signaling. In hepatocellular carcinoma, circMTO1/miR-541-5p/ZIC1, hsa_circRNA_104348/miR-187-3p/RTKN2, circRNA-SORE/miR-103a-2-5p and miR-660-3p, circZFR/miR-3619-5p/CTNNB1 pathways have been reported to modulate cell proliferation by affecting Wnt/β-catenin signaling. In gastric cancer, circAXIN1-encoded protein AXIN1-295aa/APC, circ_SMAD4/miR-1276/TCF4/CTNNB1, cir-ITCH/miR-17/ITCH, circCNIH4/DKK2/FRZB axis have been confirmed to regulate proliferation by targeting Wnt/β-catenin signaling pathway. In colorectal cancer, circ-ACAP2/miR-143-3p/FZD4 axis, circAGFG1/miR-4262 and miR-185-5p/YY1 and CTNNB1, circ5615/miR-149-5p/TNKS, hsa_circ_0009361/miR-582/APC2 pathway were reported to influence proliferation via modulating Wnt/β-catenin signaling.

Wnt/β-catenin pathway is another signaling pathway related to tumor progression, which can be regulated by circRNAs through multiple mechanisms under different settings (Fig. 8). For instance, in the context of lung cancer, circ-EIF3I facilitates cell proliferation by targeting miR-1253/NOVA2 to regulate Wnt/β-catenin pathway; circVAPA promotes cell proliferation by targeting miR-876-5p/WNT5A to activate Wnt/β-catenin signaling; circ-ZNF124 enhances cell proliferation via miR-498/YES1 axis to activate Wnt/β-catenin signaling pathway; cir-ITCH suppresses tumor proliferation by serving as the sponge of miR-7 and miR-214 to upregulate ITCH and then inactivate Wnt/β-catenin pathway [179–182]. In the context of hepatocellular carcinoma, circMTO1 suppresses tumor proliferation by regulating miR-541-5p/ZIC1 axis to inactivate Wnt/β-catenin signaling pathway; hsa_circRNA_104348 promotes cell proliferation through miR-187-3p/RTKN2 axis to activate Wnt/β-catenin pathway; circRNA-SORE functions as the sponge of miR-103a-2-5p and miR-660-3p, thus competitively activating Wnt/β-catenin signaling; circZFR enhances proliferation by regulating miR-3619-5p/CTNNB1 to activate Wnt/β-catenin signaling pathway [183–186]. In gastric cancer, circAXIN1 promotes cell proliferation by encoding a protein named AXIN1-295aa, which competitively interacts with APC and then activates the Wnt/β-catenin signaling pathway; circ_SMAD4 facilitates cell proliferation and gastric carcinogenesis by targeting miR-1276/TCF4/CTNNB1 axis to activate Wnt/β-catenin pathway; cir-ITCH inhibits cell proliferation by sponging miR-17 to increase linear ITCH expression and thus negatively regulate the Wnt/β-catenin pathway; circCNIH4 inhibits cell proliferation through enhancing DKK2 and FRZB expression and then suppressing Wnt/β-catenin signaling pathway [187–190]. Under the setting of colorectal cancer, circ-ACAP2 facilitates cell proliferation through modulating miR-143-3p/FZD4 axis to activate Wnt/β-catenin pathway; circAGFG1 accelerates cell proliferation and tumor growth by directly sponging miR-4262 and miR-185-5p to enhance YY1 or CTNNB1 expression and then activate Wnt/β-catenin pathway; hsa_circ_0005615 (circ5615) promotes cell proliferation and arrest cell cycle through functioning as a miR-149-5p sponge to release TNKS and activate Wnt/β-catenin pathway; hsa_circ_0009361 suppresses cell proliferation by acting as a miR-582 sponge to upregulate APC2, thus inhibiting the Wnt/β-catenin signaling [191–194].

**Fig. 9:**
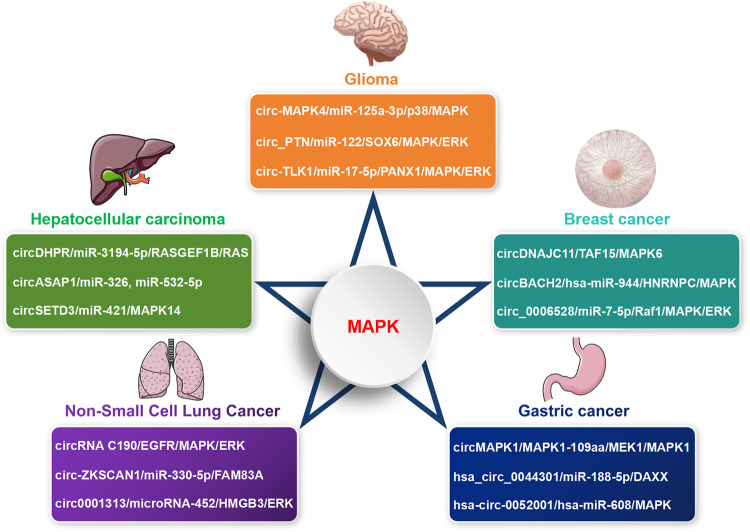
CircRNAs target MAPK signaling to regulate tumor cell proliferation and metastasis. The circRNAs involving in MAPK signaling pathway to regulate cell proliferation under the settings of various tumors, such as circ-MAPK4/miR-125a-3p/p38/MAPK, circ_PTN/miR-122/SOX6/MAPK/ERK, and circ-TLK1/miR-17-5p/PANX1/MAPK/ERK axis in glioma; circDHPR/miR-3194-5p/RASGEF1B/RAS/MAPK, circASAP1/miR-326 and miR-532-5p/MAPK1, circSETD3/miR-421/MAPK14 pathway in hepatocellular carcinoma; circDNAJC11/TAF15/MAPK6, circBACH2/hsa-miR-944/HNRNPC/MAPK, and circ_0006528/miR-7-5p/Raf1/MAPK/ERK pathway in breast cancer; circRNA C190/EGFR/MAPK/ERK, circ-ZKSCAN1/miR-330-5p/FAM83A/MAPK, and circ0001313/microRNA-452/HMGB3/ERK/MAPK axis in NSCLC; circMAPK1- encoded protein MAPK1-109aa/MEK1/MAPK1, hsa_circ_0044301/hsa-miR-188-5p/DAXX, and hsa-circ-0052001/hsa-miR-608/MAPK pathway in gastric cancer.

The mitogen-activated protein kinase (MAPK) signaling pathway is a series of highly conserved enzymatic reaction cascades involved in various physiological activities including cell proliferation, differentiation, and apoptosis. In glioma, circ-MAPK4 affects cell proliferation and apoptosis by functioning as a miR-125a-3p sponge to activate p38/MAPK signaling pathway [195]; circular RNA Pleiotrophin (circ_PTN) promotes proliferation and carcinogenesis through modulating miR-122/SOX6 axis to activate MAPK/ERK pathway [196]; circ-TLK1 facilitates cell proliferation and tumor growth by sponging miR-17-5p to target PANX1/MAPK/ERK axis [197]. In hepatocellular carcinoma, circDHPR facilitates tumor growth and metastasis through interacting with miR-3194-5p to target the RASGEF1B/RAS/MAPK signaling pathway [198]; circASAP1 promotes cell proliferation and invasion via acting as the sponge of miR-326 and miR-532-5p to regulate MAPK1 expression [199]; circSETD3 inhibits tumor growth through targeting miR-421/MAPK14 signaling pathway [200]. In breast cancer, circDNAJC11 promotes cell proliferation and tumor progression by interacting with TAF15 to upregulate MAPK6 expression and activate the MAPK signaling [201]; circBACH2 (hsa_circ_0001625) promotes cell proliferation via regulating hsa-miR-944/HNRNPC axis and stimulating MAPK signaling pathway [202]; hsa_circRNA_0006528 (circ_0006528) promotes proliferation and growth phenotypes by sponging miR-7-5p to enhance Raf1 expression and then activating the MAPK/ERK signaling pathway [203]. During NSCLC progression, circRNA C190 promotes cell proliferation via modulating the EGFR/MAPK/ERK signaling pathway [204]; circ-ZKSCAN1 accelerates proliferation and progression through sponging miR-330-5p to upregulate FAM83A expression and subsequent inactivation of MAPK signal transduction pathway [205]; circular RNA 0001313 (circ0001313) promotes NSCLC cell proliferation and invasion through serving as a microRNA-452 sponge to regulate HMGB3/ERK/MAPK axis [206]. In gastric cancer, circMAPK1 exerts suppressive function through encoding a novel protein MAPK1-109aa, which competitively binds to MEK1 and then decreases the phosphorylation of MAPK1, thus inactivating MAPK1 and the downstream molecules in MAPK signaling pathway [207]; circRNA hsa_circ_0044301 promotes cell proliferation by acting as hsa-miR-188-5p sponge to upregulate DAXX (ERK1/2) expression and MAPK signaling pathway [208]; hsa-circ-0052001 promotes cell proliferation and invasion via sponging hsa-miR-608 to regulate the MAPK pathway [209] (Fig. 9). Meanwhile, there were many circRNAs involved in the regulation of other pathways to affect tumor development. Therefore, it was an approach worthy of attention to influence circRNAs, then modulate cancer-related signaling pathways, and ultimately affect tumor progression.

### CircRNAs involvement in cell death

Cell death is divided into two categories based on how quickly it occurs and whether a drug or gene is likely to have an effect on it: accidental cell death (ACD) and regulated cell death (RCD). ACD is an uncontrolled cell death process that is triggered by unintended noxious stimuli that exceed the regulatable capacity of the cell, leading to the onset of cell death. RCD is mediated by signal transduction pathways and well-defined mechanisms of action, and plays a crucial role in homeostasis maintenance and disease development. According to different morphological, biochemical, immunological and genetic characteristics, RCD can be divided into two categories: apoptotic and non-apoptotic. Non-apoptotic RCD can be subdivided into autophagy, pyroptosis, ferroptosis, and necroptosis [210].

Apoptosis is the most classic form of cell death. A transcriptomic study using deep RNA sequencing identified differentially expressed circRNAs in HeLa cells under apoptotic conditions and manifested the potential regulatory role of circRNAs in apoptosis [211]. A well-studied circRNA derived from the tumor suppressor gene Foxo3, termed as circFoxo3 (hsa_circ_0006404), has been investigated to promote tumor cell apoptosis through various mechanisms in multiple types of human cancers [212]. In glioma, circ-MAPK4 (has_circ_0047688) was reported to inhibit cell apoptosis by functioning as miR-125a-3p sponge to regulate p38/MAPK signaling pathway [195]. It was also reported that circ-TTBK2 could act as miR-217 sponge to target HNF1β/Derlin-1 pathway and inhibit cell apoptosis in the glioma progression [213].

Autophagy is a self-digestive process that helps maintain the nutrient and energy needed by cells to limit damage in the short-term, but in the long-term it transforms into a self-destructive process that eventually leads to cell death [214]. Mounting evidence reveals that circRNAs can affect the biological behavior and treatment response of many tumors through the epigenetic regulation of autophagy [215]. Researchers found that an exosomal circRNA derived from ATG4B (circATG4B, hsa_circ_0007159) was increased in the exosomes secreted by drug-resistant colorectal cancer cells. Mechanistically, circATG4B encoded a novel protein (circATG4B-222aa), which could act as a decoy to competitively bind to TMED10 and prevent the interaction of TMED10 and ATG4B, contributing to promoted autophagy and inducing chemoresistance [216]. In pancreatic cancer, an autophagy-associated circRNA circ-autophagy related 7 (circATG7, has_circ_0064288) was reported to facilitate cell autophagy, proliferation and invasion through miR-766-5p/ATG7 and HUR/ATG7 axis, thereby promoting PC progression [217]. Furthermore, circCDYL (hsa_circ_0008285) was reported to augment autophagy of breast cancer cells via regulating the miR-1275-ATG7/ULK1 axis, which may be a potential biomarker for predicting the prognosis of breast cancer patients [218].

Pyroptosis is a newly discovered mode of programmed cell death that results in rapid cell lysis and the release of inflammatory substances, and is closely related to the progression of various cancers [219]. In lung adenocarcinoma (LUAD), circPIBF1 binds to nuclear factor erythroid 2-related factor 2 (Nrf2), which further promotes SOD2 expression and recruits EP300 to promote H3K27ac modification of SOD2, thus modulating the Nrf2/Keap-1 pathway and participating in pyroptosis-related carcinogenesis process [220]. Similarly, hsa_circ_0007312 (circ7312) was discovered to suppress lung adenocarcinoma cell pyroptosis and apoptosis via targeting the miR-764/MAPK1 axis [221]. Researchers discovered that circNEIL3 (hsa_circ_0001460) might mediate radiation-induced pyroptosis by sponging miR-1184 to upregulate PIF1 expression, providing a novel treatment option for lung adenocarcinoma therapy [222]. During the progression of ESCC, a mitochondria localized circular RNA derived from PUM1 (hsa_circ_0011240), termed as circPUM1, has been shown to play a scaffolding role in UQCRC1 and UQCRC2 interactions. Furthermore, knockdown of circPUM1 inhibited cellular oxidative phosphorylation levels, leading to pyroptosis of ESCC cell lines [223].

Ferroptosis is an iron-dependent programmed cell death manner and crucial tumor inhibition pathway, which is induced by the disturbance of intracellular lipid metabolism and accumulation of reactive oxygen species (ROS) on the membrane lipid [224, 225]. It has been demonstrated that a series of circRNAs can regulate ferroptosis via different mechanisms and then participate in cancer onset and development processes [226]. For instance, circSCN8A (hsa_circ_0026337) was found to be downregulated in NSCLC and induce cell ferroptosis through serving as a miR-1290 sponge to activate long-chain acyl-CoA synthetase-4 (ACSL4) expression [227]. In the context of bladder cancer, circST6GALNAC6 has been shown to bind to small heat shock protein 1 (HSPB1) and block erastin-induced phosphorylation site of HSPB1, protectively in response to ferroptosis stress (Ser-15 site), thus activating the P38 MAPK pathway and facilitating cell ferroptosis [228]. Additionally, researchers found that exosomal circRNA_101093 (cir93) could bind to fatty acid-binding protein 3 (FABP3) and increase its expression, desensitizing lung adenocarcinoma cells to ferroptosis [229].

### CircRNAs involvement in inflammation

Inflammation is essentially a defensive response of the human body, which is a series of complex physiological reactions produced by the immune system for the protective purposes after the body is subjected to harmful stimuli. Inflammation can be divided into acute and chronic, among which acute inflammation is the initial response of the human body to stimuli, and its ideal outcome is timely regression until the body returns to homeostasis, which help to clear inflammatory substances and promote tissue repair. In this process, immune cells and the chemicals produced by them, such as antibodies and cytokines, are released into the blood or affected tissues to fight against foreign pathogens or inflammatory stimuli. The regression of inflammation is not a passive termination of the inflammatory response, but an active programmed process initiated in the first few hours after the onset of inflammation, involving multiple types of cells, anti-inflammatory factors and pro-regression mediators. When inflammatory factors persist, inflammation cannot subside in a timely manner, and it will transform into chronic inflammation. Chronic inflammation will produce a large number of inflammatory mediators, induce cell proliferation, DNA damage and repair disorders, ultimately leading to cancer. At present, research has determined that inflammation is closely related to the occurrence and development of cancer, and it can serve as a double-edged sword to promote or inhibit tumor progression. Moreover, the inflammation response triggered by anti-tumor therapy can in turn affect the clinical therapeutic efficacy of cancer to varying degrees [230, 231].

Growing evidence has demonstrated that circRNAs can modulate the interplay between inflammation and tumorigenesis through various regulatory mechanisms, such as sponging miRNAs, binding to RBPs, and encoding proteins to regulate inflammatory signaling pathways involves in cancer progression, including NF-κB, PI3K-AKT, MAPK, JAK-STAT, etc [232, 233]. For instance, circ-TPGS2 promotes tumor-associated inflammation, cell dissemination and metastasis of breast cancer through serving as a miR-7 sponge to increase TRAF6 expression, contributing to p65 phosphorylation and nuclear translocation, ultimately inducing the activation of NF-κB signaling pathway and dysregulation of tumor microenvironment [234]. During breast cancer progression, hsa_circRNA_002178 activates inflammation and accelerates tumor growth by sponging miR-328-3p to upregulate COL1A1 expression [235]. In the context of colitis-associated colorectal carcinoma (CAC), PRKAR2A-derived circRNA (mmu_circ_0001109) exacerbates the colitis progression through activating the JAK-STAT3 and NF-κB signaling pathways, and promotes the transition of inflammation to carcinoma via activating the Wnt/β-catenin signaling pathway [236]. In addition, circNEIL3 contributes to pyroptosis and activates AIM2 inflammasome by sponging miR-1184 to release the suppressed PIF1 in lung adenocarcinoma [222].

### CircRNAs involvement in angiogenesis

One of the mechanisms closely related to the growth and metastasis of cancer is angiogenesis. Angiogenesis is necessary for tumors to obtain nutrients to grow and perform essential functions [237, 238]. It was reported that vascular endothelial growth factor (VEGF), angiopoietin, matrix metalloproteinases (MMPs), and fibroblast growth factor (FGF) could function as pro-angiogenic factors in cancer angiogenesis [239]. Numerous studies have confirmed that circRNAs mediated regulation of tumor angiogenesis [240]. For instance, circRNA-MYLK was found to function as miR-29a sponge to activate VEGFA/VEGFR2 and downstream Ras/ERK pathway to promote angiogenesis and metastasis of bladder cancer [241]. Circ3823 (hsa_circ_0001821) derived from PVT1 gene may promote colorectal cancer growth, metastasis and angiogenesis through targeting the miR-30c-5p/Tcf7 axis [242]. CircHIPK3 (hsa_circ_0000284) could function as the sponge of miR-558 to negatively regulate the expression of heparanase (HPSE), further inhibiting angiogenesis of bladder cancer cells [243]. Researchers also discovered a novel oncogenic circRNA circ-ASH2L that could act as a sponge for miR-34a to upregulate Notch 1 expression to promote tumor angiogenesis in pancreatic ductal adenocarcinoma [244].

Exosomal circRNAs also affect tumor angiogenesis by interacting with epigenetic regulators, acting as miRNA and RNA-binding protein (RBP) sponges. For example, exosomal cholangiocarcinoma (CCA)-associated circular RNA 1 (circ-CCAC1) derived from ERBB2 gene was discovered to sponge miR-514a-5p to upregulate Yin Yang 1 (YY1), inducing CCA angiogenesis, and regulate CCA tumorigenesis and metastasis [245]. Exosomal circSHKBP1 (hsa_circ_0000936) was upregulated in gastric cancer tissues and serum, but significantly decreased after gastrectomy. Functional experiments in vitro and in vivo showed that enforced expression of circSHKBP1 strengthened the proliferation, migration, invasion and angiogenesis capacities of gastric cancer. Mechanically, circSHKBP1 could sponge miR-582-3p to activate the HUR/VEGF signaling axis, and directly interact with HSP90 to disrupt the binding of HSP90 and STUB1, further restraining the ubiquitination and degradation of HSP90 [246].

### CircRNAs involvement in tumor microenvironment

Tumor microenvironment (TME) is the internal environment in which tumor cells produce and survive, including tumor cells and their surrounding fibroblasts, immune and inflammatory cells, as well as the interstitial cells, microvessels, and biomolecules infiltrating in them [247]. Classical theory holds that cancer-causing mutations in malignant cells lead to the development of cancer [248]. Subsequently, surrounding untransformed cells are recruited and adapted, accompanied by the release of various intercellular communication factors, including cytokines, chemokines, and vesicles. Some studies have shown that chronic inflammation or wound healing processes activate cancer-causing signals in abnormal microenvironment and promote tumorigenesis [249, 250].

Increasing studies have shown that circRNAs are involved in various biological regulation of TME [251]. For example, overexpression of circSMARCC1 can promote CD163 expression in macrophages via the CCL20-CCR6 axis, inducing tumor-associated macrophages (TAMs) infiltration and M2 polarization, thus contributing to the progression of prostate cancer [252]. In addition, circNEIL3-overexpressed glioma cells drive macrophage infiltration into the TME. At the same time, circNEIL3 can be packaged into exosomes by binding to hnRNPA2B1 and delivered to infiltrating TAMs, allowing them to obtain immunosuppressive properties by stabilizing IGF2BP3, which in turn facilitates glioma progression [253].

### CircRNAs involvement in other hallmarks of cancer

In addition to the major cancer features described above, circRNAs’ role in regulating other cancer hallmarks such as genome instability and metabolic reprogramming has also been discovered. The metabolic characteristics of tumor cells change during cancer progression to meet the needs of tumor growth and maintain TME homeostasis. The Warburg effect is a major metabolic characteristic regulated by many tumor cells and oncogenes. Tumor cells mainly use glycolysis pathway to provide energy under aerobic conditions, thereby improving the ability to escape apoptosis, invasion and resist chemotherapy [254, 255]. Many studies have shown that circRNAs can influence glucose metabolism in tumors through transcription factors or signaling pathways [256]. For example, CircECE1 can interact with c-Myc to prevent the speckle-type POZ protein from degrading c-Myc, thereby activating the Warburg effect of osteosarcoma via the c-Myc/TXNIP axis [257]. Highly expressed circNRIP1 in gastric cancer can act as the sponge of microRNA-149-5p to target AKT1/mTOR pathway and improve the glucose uptake capacity of gastric cancer cells [258]. In addition, circFOXP1 can interact with PTBP1 or sponge miR-370 to regulate PKLR, thus promoting the Warburg effect in gallbladder cancer progression [259].

Genome instability (GI) covers changes from a single nucleotide to the entire chromosome, contributing to the increase of genetic changes in cancer and is the driving force for cancer progression [260]. Petermann et al. showed that RNA-DNA hybridization products can be formed during transcription, DNA replication, and DNA repair, which can replace one of the DNA strands of double-stranded DNA to form an R-loop. R-loops pose a threat to genome stability and can cause DNA damage that promotes cancer progression [261]. A recent study has shown that circRNAs in the nucleus can directly regulate transcription and splicing by producing circRNAs-DNA hybrids. The team found that circRNAs enriched in mixed-lineage leukemia (MLL) recombinome and bound DNA to form a circR loop at its homologous site. These circR loops have been found to have the effect on promoting transcriptional suspension, inhibiting proteasome, chromatin translocations recombination, and DNA double-strand breaks (DSB). Together, these effects will lead to chromosomal translocations induced by endogenous RNA carcinogens in leukemia [262]. In addition, studies have shown that circRNAs, such as circBUB1B_544aa and circCHEK1_246aa, can activate the instability at the chromosomal level of multiple myeloma by phosphorylating CEP170, thus leading to the proliferation and drug resistance of tumor cells [263, 264].

### CircRNAs regulate immune checkpoints and ICIs resistance

#### CircRNAs regulate PD-1/PD-L1 in cancer

Studies have shown that circRNAs, as competing endogenous RNAs (ceRNAs), can be involved in tumor immune escape process and immunotherapy resistance, thereby regulating PD-1/PD-L1 pathway [265]. Existing studies mainly reveal the mechanisms affecting the regulation of PD-L1 expression through two approaches. One approach is that circRNAs can directly regulate the expression of PD-L1 through its sponge effect on miRNA, the other approach is that circRNAs can affect the expression of downstream molecules through the sponge mechanism, thereby modulating the expression of PD-L1. Studies on the regulatory mechanism of PD-1 are mainly focused on anti-PD-1 immunotherapy resistance. All mechanisms were summarized in Table 3.

**Table 3:** CircRNAs regulate PD-1/PD-L1 in cancer through ceRNA mechanism.

circRNA name	circBase ID	Parental gene	Target	Cancer type	Regulation of PD-1/PD-L1	Mechanism	Reference
circCHST15	-	CHST15	PD-L1	Lung cancer	Upregulates PD-L1 expression	Functions as miR-155-5p and miR-194-5p sponge to disinhibit PD-L1 expression	[266]
circFGFR1	hsa_circ_0084003	FGFR1	CXCR4	Non-small-cell lung cancer	Promotes anti-PD-1 resistance	Sponges miR-381-3p to activate its downstream gene CXCR4, which contributes to tumor malignant progression and anti-PD-1 immunotherapy resistance	[282]
circ-CPA4	hsa_circ_0082374	CPA4	PD-L1	Non-small-cell lung cancer	Upregulates PD-L1 expression	Sponges let-7 miRNA to upregulate PD-L1 expression	[267]
circ_0000284	-	HIPK3	PD-L1	Non-small-cell lung cancer	Upregulates PD-L1 expression	Sponges miR-377-3p to upregulate PD-L1 expression	[268]
circ_001678	-	-	ZEB1	Non-small-cell lung cancer	Activates PD-1/PD-L1 pathway	Sponges miR-326 to enhance the expression of ZEB1 and activate the PD-1/PD-L1 pathway dependent immune escape to induce CD8 + T cell apoptosis	[285]
circ_0068252	hsa_circ_0068252	-	PD-L1	Non-small-cell lung cancer	Upregulates PD-L1 expression	Sponges miR-1304-5p to positively modulate the expression of PD-L1	[269]
circFOXK2	-	FOXK2	PD-L1	Non-small-cell lung cancer	Upregulates PD-L1 expression	Sponges miR-485-5p to stimulate PD-L1 expression	[270]
circ_0014235	-	-	YAP	Non-small-cell lung cancer	Upregulates PD-L1 expression	Sponges miR-146b-5p to activate YAP, which further upregulates the expression of PD-L1	[283]
hsa_circ_0003222	hsa_circ_0003222	LARP4	PHF21B	Non-small-cell lung cancer	Promotes anti-PD-1 resistance	Sponges miR-527 to upregulate the expression of PHF21B and its downstream gene β-catenin	[286]
circIGF2BP3	hsa_circ_0079587	IGF2BP3	PKP3	Non-small-cell lung cancer	Enhances PD-L1 abundance by promoting its deubiquitination	Sponges miR-328-3p and miR-3173-5p to elevate the expression of PKP3, which binds with FXR1 protein to further stabilize OTUB1 mRNA, and OTUB1 enhances PD-L1 abundance by promoting its deubiquitination	[284]
circ-VIM	-	Vimentin	PD-L1	Esophageal cancer	Upregulates PD-L1 expression	Sponges miR-124 to positively modulate the expression of PD-L1	[281]
hsa_circ_0003288	hsa_circ_0003288	-	PD-L1	Hepatocellular carcinoma	Upregulates PD-L1 expression	Sponges miR-145 to positively modulate the expression of PD-L1	[271]
hsa_circ_0046523	hsa_circ_0046523	TBCD	PD-L1	Pancreatic cancer	Upregulates PD-L1 expression	Sponges miR-148a-3p to positively modulate the expression of PD-L1	[272]
CDR1-AS	-	CDR1-AS	PD-L1	Colorectal cancer	Upregulates PD-L1 expression	Sponges microRNA-7 to increase the expression levels of CMTM4 and CMTM6, which further upregulates cell surface PD-L1 protein levels	[273]
hsa_circ_0020397	hsa_circ_0020397	-	TERT, PD-L1	Colorectal cancer	Upregulates PD-L1 expression	Sponges miR-138 to positively modulate its target genes TERT and PD-L1	[276]
hsa_circ_0136666	hsa_circ_0136666	-	PD-L1	Colorectal cancer	Upregulates PD-L1 expression	Sponges miR-497 to upregulate PD-L1 expression	[274]
circEIF3K	-	EIF3K	PD-L1	Colorectal cancer	Upregulates PD-L1 expression	Sponges miR-214 to upregulate PD-L1 expression	[275]
circ_0000052	-	AGO1	PD-L1	Head and neck squamous cell carcinoma	Upregulates PD-L1 expression	Sponges miR-382-3p to upregulate PD-L1 expression	[277]
circ_0001598	hsa_circ_0001598	-	PD-L1	Breast cancer	Upregulates PD-L1 expression	Sponges miR-1184 to upregulate PD-L1 expression	[278]
circ_0001005	hsa_circ_0001005	-	PD-L1	Bladder cancer	Upregulates PD-L1 expression	Sponges miR-200a-3p to upregulate PD-L1 expression	[279]
circPCBP2	-	PCBP2	PD-L1	Diffuse large B-cell lymphoma	Upregulates PD-L1 expression	Sponges miR-33a/b to upregulate PD-L1 expression	[280]

As a circRNA highly expressed in lung cancer, circCHST15 can directly inhibit the expression of PD-L1 by acting as miR-155-5p and miR-194-5p sponges to accelerate tumor growth and immune escape of lung cancer [266]. In addition, circCPA4 (hsa_circ_0082374) [267] and circ_0000284 [268] are highly expressed in lung cancer and could also upregulate PD-L1 expression. Both hsa_circ_0068252 (circ_0068252) [269] and circFOXK2 [270] can promote the expression of PD-L1 through sponge mechanism, activate PD-1/PD-L1 pathway-dependent immune escape, and play a pro-tumor biological role. Moreover, several circRNAs can affect the expression of PD-L1 through the sponge mechanism in various tumors, such as hepatocellular carcinoma (hsa_circ_0003288) [271], pancreatic cancer (hsa_circ_0046523) [272], colorectal cancer (CDR1-AS, hsa_circ_0136666, circEIF3K, has_circ_0020397) [273–276], head and neck squamous cell carcinoma (circ_0000052) [277], breast cancer (circ_0001598) [278], bladder cancer (circ_0001005) [279] and hematological system cancer (circPCBP2) [280]. Specifically, upregulated circVIM has been found to release its targeted inhibition of PD-L1 expression via sponging miR-124 to promote immune escape and various malignant biological behaviors of esophageal cancer [281]. Another circRNA, has_circ_0020397, was reported to be upregulated in colorectal cancer, promoting cell activity and invasion, but inhibiting apoptosis by acting as a miR-138 sponge to positively regulate its target genes telomerase reverse transcriptase (TERT) and PD-L1 [276]. In diffuse large B-cell lymphoma (DLBCL), circPCBP2 was found to be highly expressed in DLBCL tissues and cells. Functionally, circPCBP2 can inhibit its target gene PD-L1 by directly binding to miR-33a/b, thereby promoting DLBCL cell dryness and chemotherapy resistance to prednisone (CHOP) [280].

CircRNAs involved in another regulatory mechanism of PD-L1 include circFGFR1 (hsa_circ_0084003) [282], circ_0014235 [283], and circIGF2BP3 (hsa_circ_0079587) [284], and all of them are highly expressed in lung cancer. CircFGFR1 activates its downstream gene C-X-C motif chemokine receptor 4 (CXCR4) through spongy adsorption of miR-381-3p, promoting tumor malignant progression and anti-PD-1 immunotherapy resistance [282]. Circ_0014235 alleviates the inhibition of YAP by directly sponging miR-146b-5p, thereby further upregating the expression of PD-L1, thus promoting gefitinib resistance and harmful biological behavior in NSCLC [283]. Mechanism experiments demonstrated that N6-methyladenosine-modified circIGF2BP3 by METTL3 could serve as miR-328-3p and miR-3173-5p sponge to competitively elevate the expression of plakophilin 3 (PKP3), which binds with FXR1 protein to further stabilize OTUB1 mRNA, and OTUB1 enhances PD-L1 abundance by promoting its deubiquitination [284].

CircRNA_001678 (circ_001678) [285] and hsa_circ_0003222 [286] are circRNAs that regulate the high expression of PD-1 in lung cancer. CircRNA_001678 could enhance ZEB1 expression through sponging miR-326 to activate PD-1/PD-L1 pathway-dependent immune escape, induce apoptosis of CD8 + T cells, and play a pro-tumor biological role [285]. Hsa_circ_0003222 has been studied to alleviate the inhibited expression of PHF21B and its downstream gene β-catenin by acting as a molecular sponge of miR-527, thereby promoting proliferation, migration, invasion, stemness and anti-PD-L1 immunotherapy resistance [286]. According to a recent study, circSOD2 could sponge miR-497-5p to upregulate the expression of ANXA11, which contributes to tumor malignant progression and anti-PD-1 immunotherapy resistance in hepatocellular carcinoma [287].

Except for serving as the aforementioned ceRNAs, circRNAs have also been reported to regulate the PD-1/PD-L1 pathway through interacting with proteins or other unclarified mechanisms. Ge et al. found that circBART2.2 encoded by Epstein-Barr virus (EBV) could bind to the helicase domain of RBP RIG-I in nasopharyngeal carcinoma (NPC) cells, and then activate the transcription factors IRF3 and NF-κB, resulting in enhanced expression of PD-L1 to induce tumor immune escape [288]. In addition, the circMGA/HNRNPL complex has also been found to significantly inhibit the growth of bladder cancer by stabilizing CCL5 and exerting synergistic effects with anti-PD-1 [289]. A recent publication demonstrated that heat shock protein 90 alpha (HSP90A) derived circHSP90A was significantly upregulated in NSCLC and could facilitate cell growth, stemness, and immune evasion by recruiting ubiquitin specific peptidase 30 (USP30) to further stabilize HSP90A protein, which then activates the STAT3 signaling pathway. Moreover, circ-HSP90A has also been shown to modulate the PD-1/PD-L1 checkpoint in this context by sponging miR-424-5p to target PD-L1 expression [290]. Hsa_circ_0000190 has also been reported to accelerate the tumor progression and immune evasion of NSCLC through increasing soluble PD-L1 expression, and its high expression could serve as an effective indicator for anti-PD-L1 immunotherapy resistance and poor prognosis of NSCLC patients [291]. CircCORO1C, which was highly expressed in hepatocellular carcinoma, could upregulate the expression of c-Myc and COX-2 by stimulating the NF-κB pathway and enhancing P65 phosphorylation to induce PD-L1 expression, thus promoting immune escape and tumor growth [172].

### CircRNAs regulate CTLA-4 in cancer

Compared with PD-1/PD-L1, there is relatively little research on the regulation of CTLA-4 by circRNAs, which may be due to the fact that most CTLA-4 targeting ICIs are immature and have no effective approved clinical transformation results. A total of three circRNAs have been studied to participate in the regulation of CTLA-4 (Table 4). CircUBAP2 (hsa_circ_0007367) was shown to repress antigen presentation and induce immune escape in pancreatic adenocarcinoma (PAAD) through sponging miR-494 to upregulate CXCR4 and ZEB1 proteins, then leading to increased expression of CTLA-4 and PD-1 [292]. CircWDR25 (hsa_circ_004310) and circQSOX1 (hsa_circ_0015497) could also promote the increased expression of CTLA-4 and PD-L1 through the sponge mechanism in hepatocellular carcinoma and colorectal cancer, respectively, thus driving tumor immune escape and promoting cancer progression [293, 294]. In these studies, we found that circRNAs can affect the expression of CTLA-4 and PD-L1, and whether the regulation of specific circRNAs can achieve the same therapeutic effect as the combined application of anti-CTLA-4 and anti-PD-L1 ICIs in clinical practice is the direction of future research.

**Table 4:** CircRNAs regulate CTLA-4 and other immune checkpoints in cancer.

circRNA name	circBase ID	Parental gene	Target	Cancer type	Regulation of immune checkpoints	Mechanism	Reference
circQSOX1	hsa_circ_0015497	QSOX1	PGAM1	Colorectal cancer	Promotes resistance to anti-CTLA-4 therapy	Functions as miR-326/miR-330-5p sponge to upregulate PGAM1 expression	[294]
circ-UBAP2	hsa_circ_0007367	UBAP2	CXCR4, ZEB1	Pancreatic adenocarcinoma	Increases expression of CTLA-4 and PD-1	Sponges miR-494 to upregulate CXCR4 and ZEB1 proteins, which then lead to increased expression of CTLA-4 and PD-1	[292]
circWDR25	hsa_circ_004310	WDR25	ALOX15	Hepatocellular carcinoma	Increases expression of CTLA-4 and PD-L1	Functions as miR-4474-3p sponge to upregulate ALOX15 expression	[293]
circUHRF1	hsa_circ_0048677	UHRF1	TIM-3	Hepatocellular carcinoma	Increases expression of TIM-3, and promotes resistance to anti-PD-1 immunotherapy	Sponges miR-449c-5p to upregulate TIM-3 expression in NK cells	[295]
circRERE	hsa_circ_0009581	RERE	CD47	Multiple myeloma	Increases expression of CD47	Sponges miR-152-3p to upregulate the expression of CD47	[297]
hsa_circ_0000276	hsa_circ_0000276	TRIM22	CD47, LDHA, PDIA3, and SLC16A1	Cervical cancer	Increases expression of CD47	Sponges miR-154-5p to upregulate the expression of CD47, LDHA, PDIA3, and SLC16A1	[298]

### CircRNAs regulate other immune checkpoints in cancer

Analogous with CTLA-4, there have been few studies on the regulation by circRNAs of other newly discovered immune checkpoints in recent years, such as TIM-3, SIRPα and CD47, which may be due to the fact that the mechanism of these immune checkpoints is not yet fully elucidated, and the exploration of ICIs targeting them as cancer immunotherapy is still at an early stage. As for the regulation of TIM-3, Zhang et al. reported that under the setting of hepatocellular carcinoma, circUHRF1 upregulated the expression of TIM-3 in NK cells by sponging miR-449c-5p, and inhibited the secretion of IFN-γ and TNF-α by NK cells, thus promoting tumor immune escape [295]. Another recently studied immune checkpoint, CD47, is a transmembrane immunoglobulin that is expressed on the surface of traditional human cells as a ligand for SIRPα and is overexpressed on a variety of tumor cells. The formation of complexes with CD47 and SIRPα transmits an antiphagocytic signal to macrophages, and the CD47-SIRPα pathway serves as an innate immune checkpoint to function as a druggable target, which enables cancer cells to escape from phagocytosis mediated by macrophages [296]. The impact of circRNAs targeting CD47 on cancer has been reviewed in the reference [28], which mentions that circRERE (hsa_circ_0009581) promotes the resistance to chemotherapy drug bortezomib (BTZ) in multiple myeloma (MM) via sponging miR-152-3p to upregulate the expression of CD47 [297]. Besides that, hsa_circ_0000276 could also regulate the expression of CD47 in cervical cancer and participate in the regulation of immune infiltration and trigger carcinogenic function [298]. These studies suggest that since circRNAs can influence tumor resistance by modulating novel immune checkpoints, circRNAs may be useful as biomarkers to help predict the effectiveness of ICIs. At the same time, circRNA targeting therapy may also achieve simultaneous regulation of multiple immune checkpoints.

## Conclusion and perspective

Over the past decade, ICIs have been developed and approved for clinical use in multiple cancer types at an unprecedented rate. Despite tremendous progress, ICIs have not completely solved the problem of cancer treatment. Besides discovering biomarkers to predict the efficacy and safety of ICIs, more immune checkpoint molecules may need to be identified in the next decade to provide more options for immunotherapy and bring treatment opportunities to more patients, which depends on our insights into the mechanisms of immune checkpoint regulation. This review summarizes the status quo of frequent immune checkpoints and ICIs commonly applied in cancer, such as anti-PD-1/PD-L1, anti-CTLA-4, anti-LAG-3, anti-TIM-3, and other immune checkpoints targeted mAbs. The regulatory mechanisms of immune checkpoints are complex and unclear, which stem from its complicated and diverse influencing factors, involving ncRNAs, proteins, and other elements. As a class of abundant component of the human transcriptome involved in cancer hallmarks, ncRNAs are closely associated with the expression regulation of immune checkpoints. In view of the structure’s stability, circRNAs present significantly greater advantages and potential as diagnostic and prognostic biomarkers for cancer management than other types of ncRNAs. Also, circRNAs have been confirmed to involve in various cancer hallmarks including proliferation, cell death, inflammation, angiogenesis, invasion and metastasis through functioning as miRNA sponge, interacting with proteins, encoding proteins, or other unclarified mechanisms.

In this present review, we emphasized on the molecular mechanisms by which circRNAs interfere with the therapeutic strategy of ICIs by regulating immune checkpoint pathways. PD-1/PD-L1, CTLA-4, TIM-3, CD47/SIRPɑ, and other immune checkpoints have been shown to be regulated by circRNAs, mostly through serving as ceRNAs, and sometimes through interacting with proteins or encoding proteins. Meanwhile, we found that circRNAs may regulate the expression of two or more immune checkpoints at the same time, and when the tumor developed resistance to one immune checkpoint inhibitor, circRNAs may resist the development of resistance by regulating the other immune checkpoint. Therefore, in future studies, the mechanisms by which circRNAs regulate the interaction of multiple immune checkpoints to affect the resistance of ICIs, and the question of other adverse reactions that circRNAs may cause as well as resistance to ICIs are the main directions of research. In addition, the exact contribution of circRNAs-mediated epigenetic regulation of immune checkpoints and resistance to cancer immunotherapy have not been clearly elucidated. Many critical questions about the precise molecular mechanisms of circRNAs in regulating immune checkpoints and mediating resistance to ICIs therapy remain to be resolved. For instance, considering the abundant expression of circRNAs in tumor cells and immune cells, can they directly serve as immune checkpoints to regulate tumor immunity? Is resistance to ICIs therapy mediated by circRNAs in tumor cells or immune cells, or a combination of both? Can we design synthetic RNAs specifically targeting circRNAs to regulate the cascade reactions involved in immune checkpoint pathway, thus achieving the development of new potential cancer immunotherapy regimens? Moreover, hyperprogression is a new challenge in the treatment of ICIs, and its mechanism is not yet clear. Current studies have shown that important causes of hyperprogression may include overactivation or mutation of cancer related genes or pathways in tumor cells themselves, such as the amplification of MDM2/MDM4 and EGFR mutations [299, 300], and changes in immune cells in the TME, such as increased number of regulatory T cells and M2-like differentiation of TAMs [301, 302]. However, the role of circRNAs in this process still needs to be further studied.

At present, various treatment regimens such as chemotherapy, radiotherapy, anti-angiogenic therapy targeting VEGF, interleukin therapy, epigenetic modulators, and protein kinase inhibitors have been proven to boost the body’s anti-tumor immunity. The reasonable combination application of these treatment approaches with ICIs will improve the current status of tumor immunotherapy. In addition, tumor vaccines have become the most promising anti-tumor immunotherapy method because of their specificity, safety and good tolerance. With the development of mRNA vaccines winning the Nobel Prize, circRNA vaccine research has also made great progress [303]. The circRNA vaccine, which contains internal ribosome entry site (IRES) and open reading frame (ORF), has better safety, stability, ease of production and scalability than the first-generation mRNA vaccine [304]. In recent studies, circRNA cancer vaccines delivered by lipid nanoparticle (LNP) system have shown robust immune responses and better anti-tumor effects in mouse models [38]. However, research on the use of circRNA vaccines for tumor immunotherapy is still limited. In the future, is there a possibility of using synthetic circRNAs or circRNA inhibitors as an alternative therapy for ICIs remedy or as a combination therapy with ICIs? Recent research advances in single-cell transcriptomics, spatial transcriptomics and epigenetics may provide important information for circRNAs’ function, phenotype in cells and location in TME, and discover additional circRNAs potentially related to immune checkpoints regulation, which takes a key step forward in the right direction for our understanding progress in tumor immunity and immunotherapy resistance.
